# PGC-1*α*-Dependent Mitochondrial Adaptation Is Necessary to Sustain IL-2-Induced Activities in Human NK Cells

**DOI:** 10.1155/2016/9605253

**Published:** 2016-06-20

**Authors:** Dante Miranda, Claudia Jara, Jorge Ibañez, Viviana Ahumada, Claudio Acuña-Castillo, Adrian Martin, Alexandra Córdova, Margarita Montoya

**Affiliations:** ^1^Department of Biochemistry and Molecular Biology, Faculty of Chemical and Pharmaceutical Sciences, University of Chile, Sergio Livingstone 1007, Independencia, 8380492 Santiago, Chile; ^2^Department of Biology, Faculty of Chemistry and Biology, University of Santiago of Chile, Avenida Libertador Bernardo O'Higgins 3363, 9170022 Santiago, Chile

## Abstract

Human Natural Killer (NK) cells are a specialized heterogeneous subpopulation of lymphocytes involved in antitumor defense reactions. NK cell effector functions are critically dependent on cytokines and metabolic activity. Among various cytokines modulating NK cell function, interleukin-2 (IL-2) can induce a more potent cytotoxic activity defined as lymphokine activated killer activity (LAK). Our aim was to determine if IL-2 induces changes at the mitochondrial level in NK cells to support the bioenergetic demand for performing this enhanced cytotoxic activity more efficiently. Purified human NK cells were cultured with high IL-2 concentrations to develop LAK activity, which was assessed by the ability of NK cells to lyse NK-resistant Daudi cells. Here we show that, after 72 h of culture of purified human NK cells with enough IL-2 to induce LAK activity, both the mitochondrial mass and the mitochondrial membrane potential increased in a PGC-1*α*-dependent manner. In addition, oligomycin, an inhibitor of ATP synthase, inhibited IL-2-induced LAK activity at 48 and 72 h of culture. Moreover, the secretion of IFN-*γ* from NK cells with LAK activity was also partially dependent on PGC-1*α* expression. These results indicate that PGC-1*α* plays a crucial role in regulating mitochondrial function involved in the maintenance of LAK activity in human NK cells stimulated with IL-2.

## 1. Introduction

Human NK cells are a specialized heterogeneous population of lymphocytes of the innate immune system involved in immunosurveillance and contributing to host antimicrobial and antitumor defense reactions. These cells are able to lyse target cells spontaneously without presensitization or MHC restriction [1–3]. An equally important function of NK cells is their capacity to produce large quantities of cytokines, such as IFN-*γ*, and chemokines upon activation [1, 2, 4]. Moreover, NK cells also act as regulatory cells in the immune system, influencing other cells and responses and acting as a link between the adaptive and innate immune response. In this way, NK cells actively eliminate susceptible targets by recruiting and amplifying the inflammatory response through multiple mechanisms. On the other hand, development, survival, proliferation, and effectors functions of NK cells are critically dependent on cytokines, such as IFN-*α*, IL-2, IL-15, and IL-18 secreted by other cells of the immune system [5]. Of these cytokines, IL-2 is a pluripotent cytokine that can activate NK cells [6, 7], promote their migration within target tissues [8], and increase the secretion of cytokines and other small molecules [9]. Upon stimulation with IL-2, NK cells develop a strong cytolytic activity against target cells by increasing the number and size of cytoplasmic granules [10], augmenting the expression of effector molecules [11], and altering the surface expression of receptors [12]. Moreover, it has been described that IL-2 has the potential to restore the cytotoxicity and granular content of exhausted NK cells [13]. In this context, IL-2 has been used as an immunotherapeutic agent to promote NK cell antitumor activity [14–18]. In fact, early phases of in vivo IL-2 use showed that the antitumor response triggered by this cytokine was frequently attributable to NK cells [6, 19–21]. Therefore, IL-2 received approval from the FDA for the treatment of metastatic renal cell carcinoma, showing that in patients undergoing IL-2-based therapy and nephrectomy a higher percentage of circulating NK cells is a predictor of better response [22].

In recent years, evidence has shown that metabolism and cell signaling are tightly linked and regulate the acquisition of effector functions in different immune cells. Effector T cells increase glycolysis to support growth and proliferation [23–25] but also to support the ability to produce IFN-*γ* [26]. By contrast, naïve memory T cells and Treg increase mitochondrial metabolism for ATP synthesis [23–25]. Less is known about metabolism in NK cells, where it has been reported that mitochondrial dynamics are important for NK cell activity. It has been shown that mitochondria relocate towards the immune synapse and rapidly undergo a decrease in mitochondrial membrane potential upon contact with the target cells. Moreover, NK cytotoxicity was impaired in the presence of an ATP synthase inhibitor [4, 27]. So far, the evidence suggests that mitochondria participate in NK cell activity, possibly supplying the energy demands and participating in signaling. It is well established that, upon IL-2 treatment, NK cells develop stronger cytotoxic activity against target cells that were previously NK-resistant [28]. Moreover, IL-2-activated NK cells can serially hit multiple targets and replenish granular stock, restoring the cytotoxicity of “exhausted” NK cells [13]. In T cells, signals from IL-2 and costimulatory CD28 support the activation and expansion of T cells, increasing glycolytic metabolism [29]. Recently, it was demonstrated that NK cells activated with IL-15 increased aerobic glycolysis but also oxidative phosphorylation, in mice NK cells. Moreover, the researchers observed that bioenergetic adaptation is essential to sustain IL-15 NK cell proliferation and cytotoxic improvement [30]. However, until now nothing has been reported on mitochondrial behavior during the activation of NK cells with IL-2 and the importance of mitochondria in sustaining increased cytotoxic and secretory activity. Studies in human NK cell are of special interest in light of IL-2 cancer therapy [18] and for the new developed protocols targeting metabolic activity [31].

For mitochondrial biogenesis to occur, it is necessary to coordinate the expression of nuclear and mitochondrial genomes. Studies in the last years have revealed that mitochondrial activity is transcriptionally controlled, in part, by nuclear receptors and the peroxisome proliferator-activated receptor-*γ* coactivator 1- (PGC-1-) related protein family. This family is formed by 3 known isoforms PGC-1*α*, PGC-1*β*, and PGC-1 related coactivator (PRC) all of which act as transcriptional coactivator. These coregulators function by engaging nuclear receptors and transcriptional factors forming a multiprotein complex and regulates gene expression which ultimately modulates mitochondrial biogenesis and respiratory function. It has been shown that PGC-1*α* or PGC-1*β* null mice only exhibit mild phenotype, whereas mice bearing compound mutation of PGC-1*α* and PGC-1*β* die shortly after birth from heart failure, suggesting that both coregulators exert redundant functions, sharing roles that collectively are necessary for the postnatal metabolic and functional adaptation [32]. Several studies have suggested that PGC-1*α* is the critical cofactor necessary to activate mitochondrial biogenesis and respiration. In fact, the expression levels of PGC-1*α* are directly related to mitochondrial biogenesis activity [33–35]. Furthermore, PGC-1*α* gene expression is rapidly increased in response to different external stimuli that augment the energy demand in different tissues [34, 36, 37]. However, less is currently known about the role of PGC-1*α* in cells of the immune system. Recent studies have shown an important role of PGC-1*α* in hematopoietic recovery in response to stress stimuli, providing mitochondrial capacity for energy demand [38, 39].

In this study, we provide evidence that healthy, isolated human NK cells activated in vitro with high doses of IL-2 significantly increase the mitochondrial mass and membrane potential in a PGC-1*α*-dependent process. Moreover, we also observed that IFN-*γ* secretion induced by IL-2 is partially dependent on PGC-1*α* mRNA expression. Also, we show that cytotoxic activity is partially dependent on mitochondrial ATP generation.

Since generation of enhanced cytotoxic activity was established at 48 h of IL-2 treatment without a statistically significant increase in mitochondrial mass or membrane potential, our results also suggest that mitochondrial activity may be important to maintain other activities in activated NK cells as well.

## 2. Materials and Methods

### 2.1. NK Cell Purification and Cell Culture

This study was approved by the University of Santiago of Chile Ethics Committee. Human participants gave written informed consent. Human peripheral blood mononuclear cells (PBMC) were isolated by density centrifugation of lymphocyte concentrate obtained from buffy coats of healthy adult volunteers acquired from the blood bank of the Hospital Clínico of the Universidad de Chile over lymphocyte separation medium (Cellgro, Mediatech). Monocytes were depleted by plating them on Petri dishes for 1 h at 37°C, and lymphocytes were harvested, washed with pH 7.4 phosphate-buffered saline (PBS), and suspended in RPMI 1640 culture medium supplemented with 10% heat-inactivated fetal bovine serum (FBS), 100 U/mL penicillin, 100 *μ*g/mL streptomycin (HyClone, Thermo Scientific), and 20 *μ*g/mL gentamycin (Invitrogen) (supplemented RPMI). NK cells were further enriched using the NK cell negative isolation kit (Miltenyi Biotec) according to the manufacturer's instructions. This technique routinely produced highly purified NK cells with more than 90% CD3^−^ CD16/CD56^+^ cells confirmed by flow cytometry. These NK cells were cultured in supplemented RPMI medium plus 50 IU/mL of human recombinant IL-2 (R&D System®).

Daudi cells were used as target cells. This cell line was grown at 37°C in supplemented RPMI with 5% CO_2_. Experiments were carried out with cells in the logarithmic growth phase.

### 2.2. NK Cell Activation

Purified human NK cells were incubated for 48 and 72 h with 2000 IU/mL of IL-2 in supplemented RPMI at 37°C and 5% CO_2_ for activation. For control conditions, purified NK cells were incubated for the same duration with 50 IU/mL of IL-2 for maintenance.

### 2.3. Cytotoxicity Assay

A ^51^Cr-release assay was used to quantify NK cytotoxic activity. A quantity of 1 × 10^6^ Daudi cells were labeled by incubation in supplemented medium containing 100 *μ*Ci Na_2_^51^CrO_4_ (PerkinElmer) for 1 h at 37°C. We used 5000 ^51^Cr-labeled cells/well as target cells, and human NK cells as effectors cells, employing different E : T ratios. When indicated, effector cells were incubated with 5 *μ*M oligomycin for 30 min and washed twice with RPMI. All the conditions were tested in triplicate. After 4 h of incubation at 37°C and 5% CO_2_, 100 *μ*L of supernatant was recovered from each well and tested for release of radioactivity. The cytotoxicity percentage was calculated as follows: 100 × [(experimental release − spontaneous release)/(maximum release − spontaneous release)]. Maximum release was obtained from target cells lysed with 2% Triton X-100 (Sigma, St. Louis, MO, USA). Spontaneous release was always below 10% of maximum release.

### 2.4. siRNA Transfection Experiments and PGC-1*α* Expression Quantification by RT-qPCR

Purified NK cells were transfected with* SMART*pool® siRNA targeting PGC-1*α*, using Dharma*FECT*® 1 (Dharmacon, Thermo Fisher Scientific) according to the manufacturer's instructions. To maintain an acceptable level of cell viability we optimized the amount of siRNA at a final concentration of 50 nM enough to decrease 70% of PGC-1*α* expression after 24 h of cell culture (data not shown). Transfected NK cells were then incubated with IL-2 to perform the assays.

Total RNA was isolated with E.Z.N.A. Total RNA Kit II (Omega, Bio-Tek, Inc.), and cDNA was synthesized using RevertAid First Strand cDNA Synthesis kit (Fermentas, Thermo Fisher Scientific). Quantitative RT-PCR for PGC-1*α* was performed using Maxima SYBR Green qPCR Master Mix (Thermo Fisher Scientific). Human PGC-1*α* was amplified using the following primers: 5′-TCTCCCTGTGGATGAAGACG-3′ (forward) and 5′-GACTAGCCTCATTGTCAGTGG-3′ (reverse). We used rRNA 18S as housekeeping gene with the following primers: 5′-CGCTACACTGACTGGCTCAG-3′ (forward) and 5′-AAAGGGCAGGGACTTAATCAAC-3′. Melting curve analysis was conducted for all primers to verify that the primers resulted in a single peak of fluorescence with no primer dimers. Ct values were normalized by subtraction of Ct values for 18S rRNA. Relative expression levels were obtained with the comparative method, using the formula 2^−ΔΔCt^, and then normalized to expression levels of siRNA-treated control cells.

### 2.5. Determination of Mitochondrial Mass and Membrane Potential

For mitochondrial mass estimation, NK cells treated under the different conditions were labeled with Mitotracker® Green FM (Molecular Probes, Invitrogen), following the manufacturer's instructions. Briefly, 1 × 10^5^ cells were incubated with 50 nM of Mitotracker Green FM for 30 min at 37°C. The cells were washed twice with PBS, resuspended in cold PBS, and analyzed by flow cytometry. Analyses were performed on a FACSort (Becton-Dickinson) and analyzed with CellQuest software.

The cytofluorometric analysis of mitochondrial membrane potential was evaluated with 5,5′,6,6′-tetrachloro-1,1′,3,3′-tetraethylbenzimidazolylocarbocyanine iodide (JC-1) (Molecular Probes, Invitrogen). JC-1 accumulates in the mitochondrial matrix, and in the presence of low membrane potential it is in its monomeric form, displaying a green fluorescence with an emission wavelength of 525 nm (FL1), whereas, in the presence of high membrane potential, JC-1 shows red fluorescence with an emission length of 590 nm (FL2). Consequently, changes in mitochondrial membrane potential are determined using the FL2/FL1 ratio. NK cells incubated with 50 or 2000 IU/mL of IL-2 were labeled with probes at a concentration of 6 ng/mL for 15 min at 37°C. The cells were washed twice with PBS, resuspended in cold PBS, and analyzed by flow cytometry.

### 2.6. IFN-*γ* Quantification

Secreted IFN-*γ* was measured using IFN-*γ* Ready-Set-Go Kit (eBioscience) in accordance with the manufacturer's instructions. Quantification was performed directly from the supernatant of human NK cells incubated in the presence of 50 or 2000 IU/mL IL-2 for 72 h. Each measured sample was normalized with control siRNA-treated NK cells incubated with 50 IU/mL IL-2.

### 2.7. Statistical Analysis

 Statistical analysis was performed using GraphPad Prism 6.00 software. Significance was determined by one-way ANOVA when we compared controls to IL-2-activated groups or one-way ANOVA followed by Tukey's multiple comparison test when we compared siRNA and IL-2 treated groups.

## 3. Results

### 3.1. Activation of NK Cells with IL-2 Induces an Increase in Mitochondrial Membrane Potential and Mitochondrial Mass as well as an Increase in PGC-1*α* Expression

**Figure 1:**
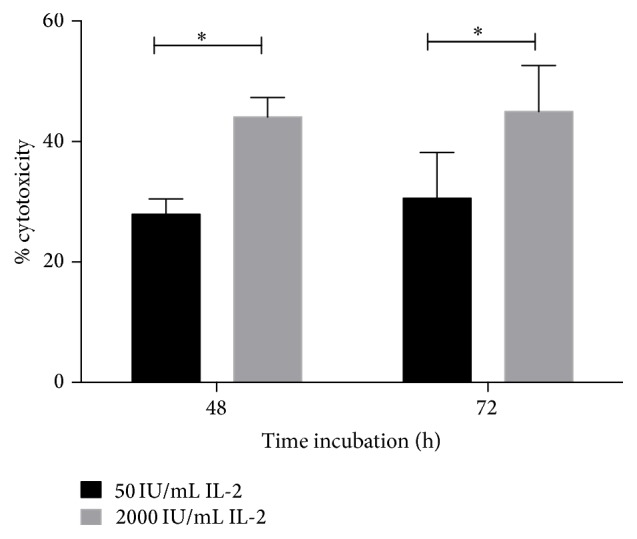
Human purified NK cells incubated with IL-2 increase cytotoxic activity. NK cells were incubated for 48 or 72 h in the presence of 50 IU/mL (control) (black bar) or with 2000 IU/mL to induce LAK activity (grey bar). Cytotoxic activity was measured in a 4 h ^51^Cr release assay using Daudi cells as target cells and an effector : target ratio of 1 : 1. Results are mean ± SE from at least 8 independent experiments (^*∗*^ *p* < 0.05).

Since we wanted to investigate mitochondrial behavior in IL-2-activated NK cells in response to IL-2, we first analyzed the effect of recombinant human IL-2 on cytotoxicity of purified NK cells. In order to maintain NK cells in culture for up to 72 h, we incubated the control cells with 50 IU/mL of IL-2 (control NK cells). As shown in Figure 1, at 48 h of treatment with 2000 IU/mL of IL-2, NK cells reach the highest cytotoxic activity, which is maintained up to 72 h of incubation. The increase in activity induced by high IL-2 concentration was approximately 30% in both times.

**Figure 2:**
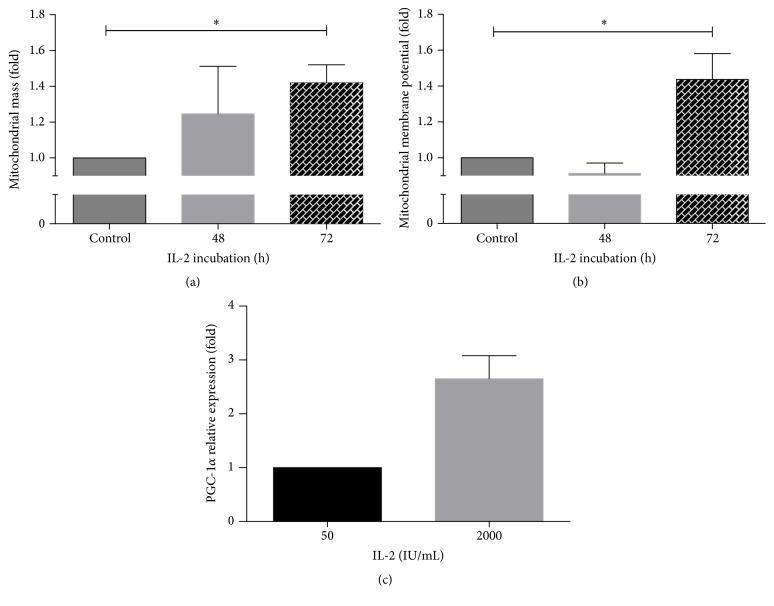
IL-2 induces an increase in mitochondrial mass, mitochondrial membrane potential, and PGC-1*α* expression in human NK cells. NK cells were cultured in the presence of 50 IU/mL (control) or 2000 IU/mL IL-2 for 48 or 72 h and then analyzed by flow cytometry to measure mitochondrial mass (a) or mitochondrial membrane potential (b) as indicated in Materials and Methods. Data are shown as the mean of fluorescence intensity in cells and normalized against controls. Results are mean ± SE from 7 independent experiments (^*∗*^ *p* < 0.05). (c) PGC-1*α* mRNA expression was determined by qRT-PCR. Relative expression was normalized with control NK cells, and results show the mean of 5 independent experiments ± SE (^*∗*^ *p* < 0.05).

It has been found that mitochondria of NK cells participate in immune synapsis, probably providing a local and rapid source of energy [27]. Also, it was demonstrated that NK cells activated with IL-15 increase their maximal respiratory capacity. As NK cells treated with IL-2 develop stronger cytotoxic activity and are able to secrete cytokines such as IFN-*γ*, it is reasonable to suppose that NK cells will require additional energy to support those activities. In order to determine if mitochondria adapted in response to IL-2 activation, we first analyzed the mitochondrial mass in NK cells treated with 2000 IU/mL of IL-2. The mitochondrial mass was relativized to the mitochondrial mass of the control NK cells. As shown in Figure 2(a), an increase in mitochondrial mass was observed at 48 h and at 72 h. However, this increase was statistically significant only at 72 h of IL-2 treatment, when the mitochondrial mass increased by 42%.

In view of the fact that IL-2 induced an increase in mitochondrial mass in NK cells, we decided to analyze the mitochondrial membrane potential as an indicator of the mitochondrial energy status. We found that NK cells showed a statistically significant increase of 44% in mitochondrial membrane potential after 72 h of IL-2 incubation (Figure 2(b)). This indicates that IL-2-activated NK cells not only have more mitochondrial mass, but also have a greater ATP producing potential. At 48 h of IL-2 treatment, the mitochondria did not show statistically significant changes in mitochondrial membrane potential or mass.

The observation that mitochondria respond to IL-2 signaling by increasing their mass prompted us to analyze mitochondrial biogenesis. All the signaling pathways involved in mitochondrial biogenesis seem to share the common key component PGC-1*α*, which activates and coordinates this process. We observed that stimulation with IL-2 (2000 IU/mL) increased the expression of PGC-1*α* in human NK cells by almost 3 times (Figure 2(c)).

### 3.2. Increase in PGC-1*α* Expression Mediates Mitochondrial Response to IL-2 Signaling

**Figure 3:**
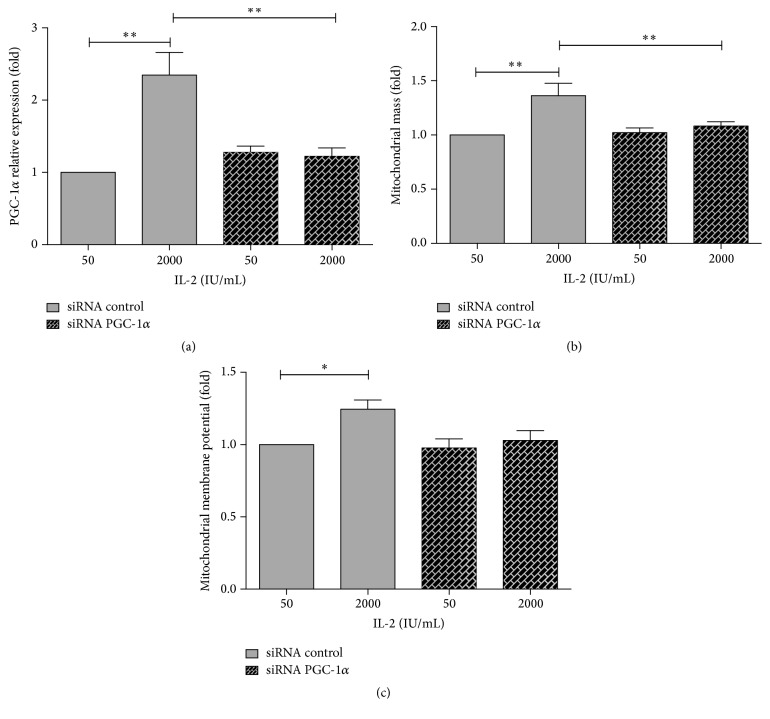
Increase in mitochondrial mass and mitochondrial membrane potential induced by IL-2 is mediated by an increase in PGC-1*α* expression. Purified human NK cells were transfected with siRNA specific for PGC-1*α* or negative controls and then treated with 50 (control) or 2000 (activated) IU/mL of IL-2 for 72 h. (a) PGC-1*α* mRNA expression was determined by qRT-PCR. Relative expression was normalized with control NK cells, and results show the mean of 8 independent experiments ± SE (^*∗*^ *p* < 0.05). (b) Mitochondrial mass. (c) Mitochondrial membrane potential was analyzed by flow cytometry. Data are shown as the mean of fluorescence intensity in cells and normalized against controls (control siRNA and 50 IU/mL IL-2). Results are mean ± SE from 5 independent experiments (^*∗∗*^ *p* < 0.01).

In order to confirm that the increases in mitochondrial mass and membrane potential were mediated by PCG1*α* upregulation, we transfected purified human NK cells with siRNA specific for human PGC-1*α* prior to IL-2 treatment. As observed in Figure 3(a), transfection with PGC-1*α* siRNA in NK cells before IL-2 activation blocked the increase in PGC-1*α* expression. Interestingly, both samples treated with PGC-1*α* siRNA do not show a significant difference in PGC-1*α* expression compared to control cells (50 IU/mL IL-2) treated with negative control siRNA.

Analyzing the mitochondrial mass in siRNA transfected cells, we first observed that IL-2 maintained the capacity to increase PGC-1*α* when we transfected NK cells with negative control siRNA. However, when we avoid the increase in PGC-1*α* expression induced by IL-2 in NK cells, we also prevent the increases in mitochondrial mass (Figure 3(b)) and mitochondrial membrane potential (Figure 3(c)). Thus, PGC-1*α* is mediating mitochondrial adaptation in response to IL-2 in human NK cells.

### 3.3. Inhibition of ATP Synthase with Oligomycin Downregulates Cytotoxic Activity in NK Cells

**Figure 4:**
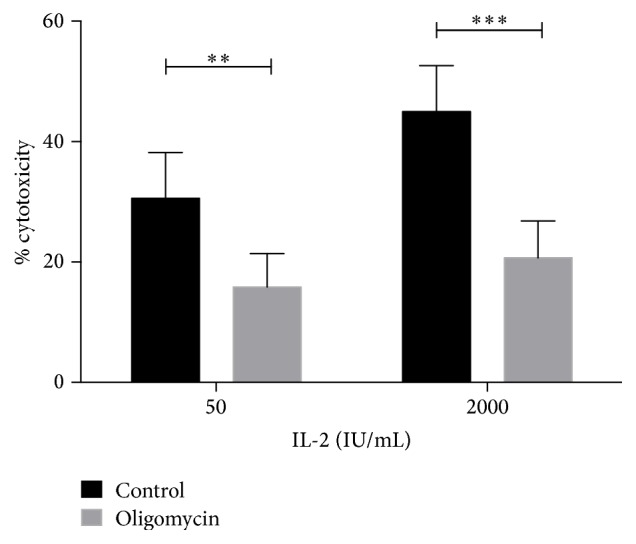
Inhibition of ATP synthase with oligomycin decreased cytotoxicity in NK cells and IL-2-activated NK cells. Human NK cells were incubated in culture for 72 h in the presence of 50 IU/mL (control) or 2000 IU/mL to induce LAK activity. When indicated, cells were pretreated with 5 *μ*M oligomycin for 30 min. Cytotoxic activity was measured in a 4 h ^51^Cr release assay using Daudi cells as target cells and a 1 : 1 effector : target ratio. Results are mean ± SE from at least 7 independent experiments (^*∗∗*^ *p* < 0.01; ^*∗∗∗*^ *p* < 0.001).

It has been reported that mitochondrial ATP synthesis is important for carrying out the NK lytic function [27]. On the other hand, we observed that NK cells treated with IL-2 increased their mitochondrial mass and membrane potential at 72 h. To evaluate whether mitochondrial ATP synthesis is important in carrying out the cytolytic activity in activated NK cells, we analyzed the cytotoxic activity in the presence of oligomycin, a specific inhibitor of ATP synthase. As expected, oligomycin decreased the NK cytotoxic activity in the control cells maintained with 50 IU/mL of IL-2 at 72 h (Figure 4). Meanwhile, in NK cells cultivated with 2000 IU/mL of IL-2, oligomycin also downregulated the activity. This effect was also observed at 48 h (data not shown) with no significant difference with respect to 72 h. In both cases, cells activated with 2000 IU/mL and treated with oligomycin demonstrated reduced cytotoxic activity compared to control NK cells.

### 3.4. IFN-*γ* Secretion Is Dependent on PGC-1*α* Expression in Human NK Cells Activated with IL-2

**Figure 5:**
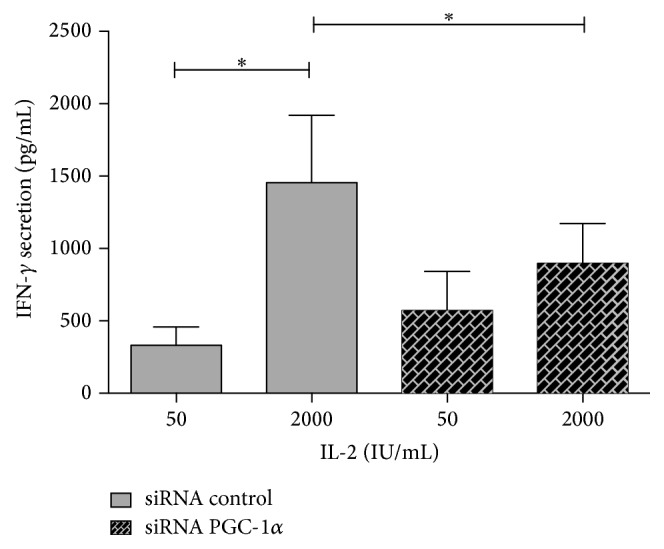
PGC-1*α* upregulation is required for optimal secretion of IFN-*γ* production in human NK cells. Quantification of IFN-*γ* was performed by ELISA. Purified human NK cells were transfected with siRNA specific for PGC-1*α* or negative control and then treated with 50 (control) or 2000 (activated) IU/mL of IL-2 for 72 h. Results show the mean of 5 independent experiments ± SE (^*∗*^ *p* < 0.05).

Another important function of NK cells is the capacity to produce IFN-*γ* when they are activated with cytokines such as IL-2.  Figure 5 shows that when human NK cells are first transfected with control siRNA and then stimulated with 2000 (IU/mL) IL-2 for 72 h, IFN-*γ* levels secreted increase five times with respect to NK cells incubated with 50 IU/mL. To examine the role of PGC-1*α* expression in the IL-2-driven IFN-*γ* secretion, we used siRNA specific for PGC-1*α* and then incubated the NK cells with 2000 (IU/mL) and 50 (IU/mL) IL-2 for 72 h. The results showed that blocking PGC-1*α* expression induced a significant inhibition of 40% in the secretion of IFN-*γ* in NK cells incubated with 2000  IU/mL of IL-2. NK cells incubated with 50 IU/mL of IL-2 secreted similar levels of IFN-*γ* whether they were incubated in the presence of siRNA PGC-1*α* or siRNA control.

## 4. Discussion

One of the main functions of NK cells is their capacity to perform cytolytic activities against transformed or infected cells. Following target recognition, NK cells activate a cascade of intracellular signals resulting in Ca^+2^ flux, polarization of granules, and subsequent directed release of cytolytic granules content against the target cell. After detachment, NK cells should restore their cytotoxic potential, generating new lytic granules and reexpressing activating receptors (reviewed in [40]).

One of the best characterized cytokines able to activate human NK cells is IL-2. Clinical trials using IL-2 have shown that the antitumor response triggered by this cytokine was frequently attributable to NK cells [7, 19–21]. It is well known that NK cells stimulated with IL-2 generate an enhanced lymphokine activated killer activity (LAK) by means of increasing the number and size of cytoplasmic granules [11] and the expression of effector molecules [12] as well as modifying the surface expression of NK receptors [13]. Moreover, IL-2-activated NK cells increased both their capacity to migrate and their capacity to contact different possible target cells [35].

To execute this effector function, NK cells should require a large amount of energy. Mounting data, primarily derived from the mouse model, indicate that metabolism and cytokine cell signaling are intimately linked and regulate the acquisition of effector functions as well as proliferation and differentiation in immune cells.

Thus, we decided to determine if human NK cells with LAK activity alter their metabolism to support the bioenergetic demand for performing cytotoxic activity more efficiently.

Given that LAK activity was established at 48 h of IL-2 incubation with no further increase but mitochondrial mass and mitochondrial potential were significantly increased after 72 h, these results suggest that mitochondrial ATP synthesis could be important in supporting, instead of developing, the LAK phenotype.

The observation that mitochondria respond to IL-2 signaling by increasing mitochondrial mass and potential prompted us to analyze mitochondrial biogenesis. All the signaling pathways involved in mitochondrial biogenesis seem to share the common key component PGC-1*α*, which activates and coordinates this process.

To further investigate the influence of PGC-1*α* expression on IL-2-mediated increases in mitochondrial mass and mitochondrial potential, we transfected HPNK cultured cells with siRNA specific for human PGC-1*α* prior to IL-2 treatment. As anticipated, the inhibition of PGC-1*α* mRNA expression resulted in an inhibition of the IL-2-mediated increase in mitochondrial mass and mitochondrial potential. This result reveals an important role that mitochondria are playing in IL-2-induced LAK activity in human HPNK cells.

Interestingly, our results show that when we used siRNA for PGC-1*α* in NK cells treated with 50 IU/mL of IL-2 a decrease in PGC-1*α* mRNA expression was not seen compared to cells with control siRNA. Moreover, PGC-1*α* siRNA-treated cell activated with 2000 IU/mL of IL-2 also showed the same PGC-1*α* expression as that in siRNA control treated with 50 IU/mL. This is in agreement with the mitochondrial mass observed for siRNA PGC-1*α* that is not different from control. These results could be explained, because given that PGC-1*α* is an important cofactor necessary to sustain mitochondrial activity and therefore energetic status, we cannot discard that siRNA-induced PGC-1*α* decreased expression forced the cell through some sort of feedback mechanism to keep PGC-1*α* expression at basal levels necessary to sustain the energy requirements for cell function. Further studies to determine the requirement of basal PGC-1*α* expression for human NK cell survival and activity will be of importance to increase the knowledge of NK cell function.

Mitochondria are highly dynamic organelles that produce most of the cellular ATP and are essential components of different signaling pathways acting as a spatial Ca^+2^ buffer [36]. Recently, it was described that IL-15, a cytokine closely related to IL-2, activates murine NK cells and that this process is coupled to a metabolic regulation. The authors describe that murine splenic NK cells have a low basal metabolism that was enhanced by IL-15 stimulation, increasing oxidative phosphorylation and glycolysis in an mTOR signaling dependent way [30]. IL-15 and IL-2 share two of the three possible receptors chains. IL-2 signals via specific receptors formed by combination of three different IL-2R subunits. Of these receptors, IL-2R*β* (CD122) and *γ*_c_ (CD132) are shared with IL-15 R and are constitutively expressed in NK cells. IL-2R*α* (CD25) is a key component to form the high-affinity heterotrimeric IL-2R*αβγ*_c_ on human NK cells. CD25 is poorly expressed in resting NK cells, but IL-2R*βγ*_c_ form an intermediate-affinity IL-2 receptor, which can signal at a higher IL-2 concentration. IL-2 activates different signaling pathways including JAK/STAT, Ras-MAP kinase, and PI3K-Akt [37], which could be involved in IL-2 dependent LAK generation.

It is known that in T cells IL-2 is a potent activator of mTORC1 activity via PI3K activation [38, 39, 41]. Upon activation, T cells rapidly become anabolic, increasing glucose uptake and glycolysis to derive energy and create biosynthetic substrates, promoting cellular growth and clonal expansion [39]. Furthermore, it was also discovered that mTORC1 controls the transcriptional activity exerted by PGC-1*α* by directly altering its physical interaction with another transcription factor, namely, yin-yang 1 (YY1) [40]. In this way, mTORC1 and YY1 are described to increase PGC-1*α* activity but not its expression. We have observed that IL-2 increased PGC-1*α* expression and we specifically blocked this increase using siRNA. Given that there is no data about possible regulation of the expression of YY-1 by PGC-1*α*, we can speculate that the IL-2 dependent increase in mitochondrial mass and potential observed in human HPNK cells takes place directly by PGC-1*α* action.

Many different pathways and stimuli have been described to increase expression of PGC-1*α*. Among these, one of the most potent activators of PGC-1*α* transcription described is the cAMP response element binding protein (CREB), which is a key transcriptional regulator of a large number of genes [42]. CREB also was previously described to be activated by IL-2 in human NK cells [43]. However, it is still unclear, and further investigation is required to elucidate the signaling pathway downstream of IL-2 involved in PGC-1*α* upregulation.

As expected, the cytotoxic activity of NK cells activated with IL-2 decreased significantly when cells were pretreated with the specific ATP synthase inhibitor oligomycin; thus a rapid supply of mitochondrial ATP is necessary to perform the cytotoxic process. However, NK cells with LAK activity retained cytotoxic activity, suggesting that the ATP needed to accomplish the cytotoxic activity is provided by glycolysis. Since IL-2 was described as a potent activator of mTOR and glycolysis, it is interesting to note that a great part of the NK cell activity seems to be dependent on mitochondrial ATP synthesis.

Another important function of NK cells is the ability to produce cytokines such as interferon-*γ* (IFN-*γ*), tumor necrosis factor-*α* (TNF-*α*), and granulocyte/macrophage colony-stimulating factor (GM-CSF) as well as chemokines under different stimuli [5, 44]. The regulatory pathways that control NK cell cytokine production are complex and not completely understood. As mentioned above, upon activation T cells switch from oxidative phosphorylation to aerobic glycolysis. Moreover, it was demonstrated that GAPDH is capable of inhibiting the translation of IFN-*γ* mRNA by binding to its 3′UTR. In this way, the ability of activated T cells to produce IFN-*γ* is dependent on GAPDH reengaging with glycolysis, thereby releasing the mRNA [26]. In NK cells, IFN-*γ* secretion might also be partially dependent on increased glycolysis. However, in this study we furthermore demonstrated that the optimal secretion of IFN-*γ* by human NK cells upon activation with IL-2 is also dependent on PGC-1*α* upregulation.

It is well known that NK cells can be activated by other cytokines like IL-15, IL-12, and IL-18, which differentially induce cytotoxicity and secretion of cytokines. For instance, IL-12 induces through STAT4 pathway release of larger amount of IFN-*γ* in comparison to IL-2. The main effect of IL-18 is to synergize with IL-12 to induce IFN-*γ* secretion. In fact, IL-18 stabilizes the IFN-*γ* mRNA through the activation of a MAPK p38 dependent pathway. Meanwhile, IL-15 is necessary for NK cell development and survival and also to enhance cytotoxicity [45, 46]. Further investigations are necessary to elucidate if these cytokines require the participation of PGC-1 cofactor to adapt NK metabolism for optimal functions.

On the other hand, NK cells can also be negatively regulated by cytokines such as IL-10 and TGF-*β*. IL-10 slightly reduces IL-2-activated cytotoxicity and IFN-*γ* secretion in human NK cells isolated from peripheral blood [47]. It has been shown that TGF-*β* possesses a potent activity in suppressing the production of IFN-*γ* in NK cells stimulated with IL-2 and IL-12 or through activated CD16 [48]. In addition, TGF-*β* signaling suppresses IL-2-induced NK cytotoxicity inhibiting the promoter-binding activities of different transcription factors such as CREB [47]. As mentioned before, CREB is one of the most potent activators of PGC-1*α* expression, so it is possible that TGF-*β* might induce its inhibitory actions by preventing PGC-1*α* expression and, as a consequence, mitochondrial metabolism adaptation of NK cells in response to IL-2. Further studies will be necessary to clarify whether TGF-*β* signaling is involved in PGC-1*α* inhibition.

## 5. Conclusion

In this study we demonstrate that IL-2 induces an increase in mitochondrial mass as well as membrane potential in human NK cells in a manner dependent on an increase of PGC-1*α* expression. Our work shows that mitochondrial dynamics are essential for supporting IL-2-activated effector functions such as LAK activity and IFN-*γ* secretion in human NK cells.
